# Rethinking pain communication of patients with Alzheimer’s disease through E-textile interaction design

**DOI:** 10.3389/fphys.2023.1248893

**Published:** 2023-11-27

**Authors:** Yanheng Li, Long Bai, Yaxuan Mao, Hongliang Ren, Yu Qiao, Xin Tong, Ray Lc

**Affiliations:** ^1^ School of Creative Media, City University of Hong Kong, Kowloon, Hong Kong SAR, China; ^2^ Department of Electronic Engineering, The Chinese University of Hong Kong, Shatin, Hong Kong SAR, China; ^3^ Department of Biomedical Engineering, National University of Singapore, Singapore, Singapore; ^4^ School of Design and Arts, Beijing Institute of Technology, Beijing, China; ^5^ Data Science Research Center and Global Health Research Center, Duke Kunshan University, Kunshan, China

**Keywords:** Alzheimer’s disease, caregiver, chronic pain, wearable sensor, multi-sensory stimulation, pain communication, tangible interaction

## Abstract

Older individuals are easily prone to chronic pain. Due to the complexity of chronic pain, most elderly often have difficulty expressing pain to others to seek assistance, especially those with Alzheimer’s disease (AD). The caregivers cannot instantly discover the patients’ pain condition and provide timely pain management. This project applies physiological signal sensing technology to help AD patients express the presence of pain non-verbally. We embed sensors on patients’ handkerchiefs to identify the patient’s abnormal physical activity when pain occurs. Next, we translate the physiological signal into qualitative light alert to send to caregivers and indicate the pain occurrence condition. Then, utilizing multi-sensory stimulation intervention, we create an electronic textile (e-textile) tool to help caregivers effectively support patients in pain. And thus to create a two-way pain communication between caregivers and the patients. Pain perception can be independent of subjective expressions and tangibly perceived by others through our textile prototype. The e-textile handkerchiefs also bring up a new guide to facilitate communication for caregivers when their patients. We contribute the design insights of building a bio-sensing and e-textile system with considering the pain communication needs, patients’ pain behaviors and preference of objects. Our e-textile system may contribute to pain communication bio-sensing tool design for special elderly groups, especially those with weakened cognition and communication abilities. We provide a new approach to dealing with the pain of AD patients for healthcare professionals.

## 1 Introduction

Alzheimer’s disease (AD) is a progressive neurodegenerative disease with insidious onset (Cummings and Cole, 2002). It clinically manifests as cognitive function declines (such as memory loss, agnosia, and visuospatial skills impairment), executive dysfunction (such as apraxia), and psychiatric symptoms (such as behavioral disorders and personality changes) (Castellani et al., 2010; Apostolova, 2016). Elderly subjects with advanced AD are always exposed to progressive medical conditions, such as pain, like other aging individuals (Inelmen et al., 2012). Almost 50% percent of AD patients suffer from chronic pain (Molton and Terrill, 2014).

Chronic pain is a degenerative disease. It is usually referred to as chronic pain if it occurs for over 3 months (Merskey, 1986). The pain will cause bad emotional experiences and even depression in the patients, and a continuously bad affective state will exacerbate the pain condition, which is one of the causes of chronic pain suffered by AD patients (Zhuo, 2016). Chronic pain is often under-treated or misdiagnosed due to its long duration and progression (Ashburn and Staats, 1999). Some patients may ignore the pain when the pain is in low intensity. They seek diagnosis only when pain gradually develops and finally find it unbearable. Due to the lack of attention to the pain, it is hard for the patients to tell accurately about the pain and their condition, while others and caregivers can hardly help them to express it as pain is a very subjective experience and invisible to others. Besides causing physical suffering, chronic pain also profoundly affects patients’ mood, personality, and social interaction, reducing their quality of life and impairing their ability to deal with daily affairs (MIleS et al., 2005). Therefore, treating chronic pain requires not only accurate assessment, but also an interdisciplinary treatment plan that includes reducing physical pain while improving mental condition. After the patients express their pain, caregivers and medical staff need to provide feedback to help patients with pain management, such as pharmacological or non-pharmacological behavioral interventions (massage, exercise assistance, etc.) (De Kok et al., 2018).

**FIGURE 1:**
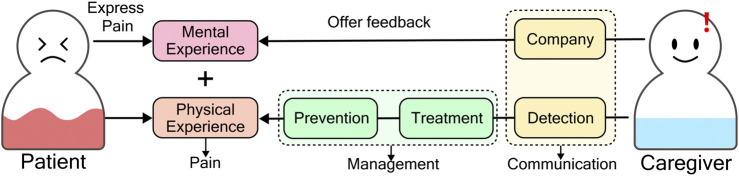
A successful two-way pain communication between patients and caregivers includes patients express pain, and caregivers offer feedback. Pain detected by caregivers means successful expression from patients. Company is the feedback from caregivers.

Pain is invisible to others, and counting the AD patients’ deterioration of communication skills is even more challenging. AD patients often cannot report their pain, and caregivers also cannot notice their condition in a timely and accurate manner, especially for those patients in advanced stages of AD. This condition will leave the patient’s pain unassessed and untreated, affecting the diagnosis of pain-causing disorders (Horgas and Elliott, 2004), which will cause sleep disturbance and agitation, causing AD patients’ emotional instability, making them feel more stressed and depressed. (McCurry et al., 1998; Garcia, 2008; Pu et al., 2020). Moreover, unintentional overlook by caregivers may also result in a sense of lack of company, which will frustrate the patients. Finally, chronic pain will aggravate and develop. The facts listed above suggested, when there is pain occurring in AD patients, as Figure 1 shows, caregivers should 1) be aware of the pain condition instantly (patients communicate their pain to caregivers from one side), 2) give immediate feedback, providing company (caregivers communicate their feedback back from the other side), and 3) helping the patients relieve pain (pain management).

To recognize the pain in AD patients, scales are widely adopted by caregivers and medical professionals, including self-reported scales and observation scales (Horgas et al., 2009), e.g., Visual Analogue Scales, Pain Assessment in Advanced Dementia (Zwakhalen et al., 2007). The self-report method is considered the golden rule of pain assessment because pain is a subjective experience (Pautex et al., 2007). However, impaired expression makes self-reporting troublesome for patients with advanced AD (Zwakhalen et al., 2006). The observation scales thus become the alternative evaluation used in patients who cannot complete the self-report. Though it is more useful in advanced AD with chronic pain, it is imperfect. The disadvantage of observational methods is that objective views of someone’s pain are sometimes biased, because the effectiveness and accuracy of judgment depends on the medical staff’s skill of sensing and interpreting non-verbal pain cues (Nygaard, 2010).

Wearable technology embedded with different bio-sensors becomes an alternative pain evaluation solution for the scales (Chen et al., 2021). The technology helps patients’ bodies “self-report” their health condition to people by measuring and recording physiological signals such as heart rate and electrocardiogram (ECG), respiratory rate, skin electricity, and physical activity in real time (Leroux et al., 2021). It can also be used for long-term monitoring and assessment of health conditions. These properties make wearable devices ideal for the non-verbal expression of pain. Wearable sensors are not only integrated into devices like the Apple Watch, Fitbit, and smartphone application (APP) to tell body data and information. They are also combined with electronic textile (e-textile) technologies, which integrate conductive materials, batteries, and electronic components (sensors, etc.) into textiles, to give the textile electronic functionalities such as sensing and communicating (Komolafe et al., 2021). The e-textile has some advantages compared with those wearable devices: first, the fabric is flexible and well adapted to the human body, which can be more comfortable and make the sensors contact directly with skin; second, fabrics are easily customizable, so that we could add various patterns with electronic functions to provide different tangible interactions, for instance, press the soft fabrics to trigger LED lights on (Amitrano et al., 2020).

Existing wearable sensor-based devices for pain recognition primarily allow patients to unilaterally report pain to caregivers, often by detecting pressure from hand movements to indicate pain levels (Böing-Meßing et al., 2022). However, these devices are not tailored to the unique needs of patients with Alzheimer’s disease (AD). Moreover, considering AD patients’ cognitive and communication limitations, our interviews have revealed that caregivers struggle to effectively soothe their pain and emotions even when informed about their discomfort. Limited research addresses the challenge of enabling two-way pain communication. Existing solutions such as RepWear (Rodríguez et al., 2017) and Weather (Tong et al., 2015) require patients to self-report pain by pressing a button on the wearable device. These devices, however, lack mechanisms for caregivers to offer feedback and interactions to patients. In this context, the application of multi-sensory stimulation (MSS) therapy through Fidget blankets, a tactile and interactive fabric-based tool designed to provide sensory stimulation and engagement for AD patients, offers an avenue for caregivers to promptly provide feedback and interaction. These blankets incorporate customizable attachments that patients can manipulate and interact with using their hands. Caregivers can guide patients to interact with the blankets, and encourage interactions that could enable effective feedback when pain is detected. Therefore, we propose the e-textile system involving bio-sensors, processing boards, and interactive fabrics as customized attachments within the blanket. This e-textile system, embedded with sensing technology to detect and convey pain information and integrated with interactive panels to allow feedback, holds the potential to establish a viable interface for two-way communication with AD patients.

Based on the states of diagnosis and treatment of chronic pain in AD patients mentioned above, this paper proposes the following research questions to help us rethink patient-caregiver relationships by applying tangible sensing e-textile technologies, and seek new approaches to help them break the barrier of verbal expression.

1) **For AD patients**: What is patients’ need for pain information communication with their caregivers? How can we help them express pain non-verbally? How to sense patient pain non-verbally using wearable sensors?
2) **For caregivers**: What is caregivers’ need for the access patient’ pain and provide feedback? How can we help to facilitate two-way communication between patients and caregivers with e-textile technology and MSS therapy effectively?

We conducted interviews with six families of AD patients and caregivers, and designed the e-textile system based on multi-sensory stimulation (MSS) intervention, the tangible interaction in our system, to assist communication about pain between patients and caregivers. We also presented a use case on evaluation and addressed the opportunities and challenges of the system. The main contributions of our work are as follows.

• We identified the most pressing communication needs when pain occurs in AD patients through case-study interviews and observations. We also outlined the design criteria for designing a close-fitting computing device to detect AD patients’ pain by observing their preference for objects and behaviors in everyday life.
• The e-textile system we proposed explored the possibility of a tangible interactive entity as a non-pharmacological intervention for pain in AD patients. Our system is not only a one-way message to the caregiver like other body data monitoring devices, and it could help the patient and the caregiver with two-way communication. Findings in our case study suggested that the patients seem calmer and more comfortable after enjoying the system with their close family when they are in pain, showing that it is important for caregivers to promptly react and accompany the patients in pain status. Moreover, the users advised us to improve our design, for example, by differing the level and complexity of interaction between the patient’s and caregiver’s sides to enhance the caregivers’ engagement.

## 2 Related works

### 2.1 Monitoring pain condition with wearable device

In recent years, with the advent of wearable smart devices and physiological signal sensing technologies, implementing ubiquitous non-invasive systems for pain identification and monitoring has become possible. In this study, the wearable bio-data technology is considered to be the objective approach for caregivers to read the patients’ pain state and an alternative self-report of the patients. Therefore, it is necessary to identify each bio-sensor’s capability and limitation, so we could choose the appropriate sensor for discerning a patient’s pain state.

Wearable devices are introduced to monitor patients’ pain long-term outside the hospital. An electrocardiogram (ECG) sensor can collect data on the surface of the chest or limbs. People usually infer whether the pain occurs by analyzing heart rate variability (HRV) (Appelhans and Luecken, 2008; Koenig et al., 2014). However, the ECG collection needs to be performed when the user is in a calm state. Otherwise, physical activity will easily cause noise to the data and result in inaccurate detection (Naranjo-Hernández et al., 2020). As we know, people may be in a state of high tension when in pain. Simultaneously, the skin conductance will increase (Loggia et al., 2011). Galvanic skin response (GSR) sensors can determine whether a person’s body is in a state of high stress by measuring skin conductance data, which has also been statistically proven to detect an individual’s pain state effectively (Lin et al., 2022). Electroencephalogram (EEG) sensors are often engaged in clinical usage (Rissacher et al., 2007). However, refined EEG sensors are complex to wear and uncomfortable to carry with patients in daily life, while the accuracy of some tiny EEG Wearable devices are introduced to monitor patients’ pain long-term outside the hospital. An electrocardiogram (ECG) sensor can collect data on the surface of the chest or limbs. People usually infer whether the pain occurs by analyzing heart rate variability (HRV) (Appelhans and Luecken, 2008; Koenig et al., 2014). However, the ECG collection needs to be performed when the user is in a calm state. Otherwise, physical activity will easily cause noise to the data and result in inaccurate detection (Naranjo-Hernández et al., 2020). As we know, people may be in a state of high tension when in pain. Simultaneously, the skin conductance will increase (Loggia et al., 2011). Galvanic skin response (GSR) sensors can determine whether a person’s body is in a state of high stress by measuring skin conductance data, which has also been statistically proven to detect an individual’s pain state effectively (Lin et al., 2022). Electroencephalogram (EEG) sensors are often engaged in clinical usage (Rissacher et al., 2007). However, refined EEG sensors are complex to wear and uncomfortable to carry with patients in daily life, while the accuracy of some tiny EEG sensors may be easily interfered with by the patients’ activities. Thus, although pain is a kind the cerebral cortex feels, EEG sensors may not be the most suitable to be embedded in a convenient wearable device. In addition, electromyography (EMG) and accelerometers are potential tools to measure physical activity (Yang et al., 2018; Leroux et al., 2021). Some studies calibrate the degree of pain by measuring the degree of muscle contraction around the painful part or on the face (Yang et al., 2019; Leroux et al., 2021). Clinicians are getting help from Usense, a wearable wristband with multiple motion sensors, to help some AD patients record abnormal motor behaviors, so that medical staff can adjust drug and non-drug interventions in a timely manner (Fleiner et al., 2016). For example, AD patients walk an average of 7% of all physical activity per day, with an average of 8,829 steps per day (SD = 7,428) in the absence of any pain and anxiety (Fleiner et al., 2016). When the patient’s number of steps exceeds this threshold to a large extent, it could basically prove that the patient is in a state of pain and restlessness.

When individuals with AD experience pain, they often exhibit physical manifestations such as abnormal walking as a means to communicate their discomfort (Husebo et al., 2011). Consequently, this study will not use ECG and EEG sensors. The implementation of EMG and GSR sensors requires consideration of the patient’s comfort and preference, as they necessitate close adherence to the skin. Given these factors, accelerometers present a more fitting alternative. They directly measure the body’s movement, which can signify pain episodes, and are unobtrusive for patients to wear.

### 2.2 Multi-sensory stimulation therapy and pain management

For people with AD, pain can provoke anxiety. If this painful emotional experience goes unnoticed or unrelieved, it will continue aggravating their pain and damage the body or mind. Therefore, numerous positive approaches to non-medicinal treatment were used to support daily pain management for AD.

Multi-sensory stimulation therapy (MSS) therapy is most commonly used to help chronic patients relieve their negative sentiments. (Siregar, 2022). Designed to stimulate the primary senses through a pleasurable sensory experience, MSS provides stress-free, entertaining tools and toys to stimulate and relax the senses of people with AD, helping them achieve a balance between the sensory-stimulating and the sensory-calming activities (Sánchez et al., 2013). These stimulating experiences are based on primary senses, which do not depend on the patient’s previous event experience and memory. The MSS therapy has been observed to be effective in alleviating psychiatric symptoms in AD. Snoezelen, as an MSS environment, provides patients with objects related to the five senses: fiber optic cables, aromas therapy, different sounds/soothing music, water columns of different colors, textured balls to touch, and projection screens in a dimly lit room (Chung and Lai, 2002). In addition to the MSS environment, sensory busy toys can be another option. The fidget blanket can provide AD patients with tailored haptic components to support their daily leisure activities that build wellbeing, as well as narrative communication with others during play (Baldwin, 2006; Souyave et al., 2013; Kroustos et al., 2016; Kroustos et al., 2016). The fidget components can be the interactive attachments on our e-textile; different functional electronic components may enrich the sensory channel, not just haptics in the system. Moreover, the caregiver should play an essential role in the process of MSS therapy, not just giving the fidget toys to patients, which inspires us to involve the caregivers in the process through the pattern design.

## 3 Formative study and design principles

Current wearable systems are not explicitly designed for AD patients with chronic pain and behavioral interventions. To better clarify the needs of the user and the contexts in which this wearable would be utilized, We invited six families to our online interview, including one late-stage and five middle-stage AD patients (all of them are advanced AD patients) and their caregivers to be our participants, as seen in Table 1.

**TABLE 1:** The information of our target user and their caregivers. Five of the patients are middle stage AD, and one of them is late stage AD. The caregivers we recruited are those have the closest relationship with the patients.

Num	Patient gender	Patient age	AD stage	Interviewed caregiver
F1	Female	72	Late Stage	Grandson
F2	Female	70–80	Middle Stage	Grandson
F3	Female	79	Middle Stage	Granddaughter
F4	Male	76	Middle Stage	Granddaughter
F5	Female	90–100	Middle Stage	Daughter
F6	Male	74	Middle Stage	Granddaughter

### 3.1 The pain information that AD patients need to convey

According to the clinical consultation process of patients with chronic pain from physicians, pain information includes the location of pain, the degree of pain, the symptoms of pain (what conditions hurt), and the characteristics of the pain. The location of chronic pain is complex in many cases. Because chronic pain does not only cause pain at the injury but spreads to other parts of the body, causing holistic pain over time (Gerwin, 2001); for example, chronic pain in knees can lead to low back pain over time (Andersen et al., 2012). Thus, ambiguous pain location is difficult to articulate for mid to late-stage AD patients. Meanwhile, Pain levels are rated using numbers in the diagnostic process. Pain symptoms and characteristics rely on subjective recall and reports, all of which require a certain level of cognitive ability. Therefore, this information is also hard to express for mid to late-stage AD patients. Moreover, the common pain conditions in AD patients are unknown.

Therefore, **the first goal of our interview was to investigate what information about patients’ pain most needed to be communicated with caregivers**. This exploratory process first engaged all the caregivers due to the inconvenience in verbal communication for AD patients, which enabled us to gain background information on the typical pain type and basic pain information of the patients, together with how they assess the pain occurring.

**FIGURE 2:**
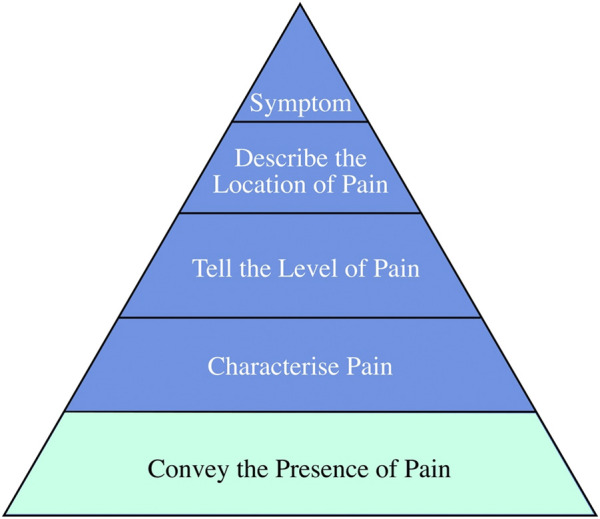
The need level of AD patients.

The typical chronic pain suggested by caregivers in AD patients “belongs” to chronic musculoskeletal pain, according to the International Association for the Study of Pain classification (Treede et al., 2015). Except F1 is a sequela of an earlier injury, and most other causes are probably aging, muscle degeneration, and the accumulation of physical activity from earlier exertion. Four caregivers (F1, F2, F3, F6) mentioned that they became aware of the pain when the patient became agitated or cried for no reason. F5’s caregiver mentioned that she could tell when the patient was in pain by facial expressions. The remaining caregiver indicated that pain is inaccessible if the patient does not communicate. All the caregivers always learned about the patients’ pain almost long after the pain had occurred. Most caregivers can determine the pain level by the intensity of the patient’s response. Regarding the pain location, F1’s caregivers indicated that they determined the location of the pain by repeated questioning, and F3’s caregivers indicated that the patient would cry out about the location of the pain—the remaining caregivers determined based on prior knowledge and experience. All the caregivers agreed that knowing the presence of pain was the most crucial information; F1’s caregiver felt that knowing the pain’s location was also necessary to facilitate subsequent medical care. Overall, letting caregivers around the patient know the pain is the most urgent need and the basis for assessing further information about pain. Drawing from the WILDA approach for clinical pain assessment Fink (2000), which emphasizes the systematic evaluation of pain characteristics, intensity, location, duration, and associated symptoms, we have structured pain information that patients may convey into five distinct levels, as illustrated in Figure 2. It is essential to underscore that recognizing the onset of pain is foundational to its assessment; hence, the primary objective is to alert caregivers to its presence.

### 3.2 Observing abnormal behavior for pain detection

Since the functions of the bio-sensors and the signals they can collect about pain have been well clarified in Section 2, **the second goal of our interview was to explore how to leverage the ability of bio-sensors in designing a system for detecting pain**. Since most AD patients were cared for in a domestic environment, the abnormal physical activities of AD patients when they were in pain, decided how the design could take effect. Therefore, we adopted the patient journey approach in interviewing the caregivers to observe and record the regular physical activities of AD patients to investigate what is abnormal and what can be captured by the sensor when they are in pain. We asked the caregivers to record and report the daily activities to us, and then we could summarize a daily activities journey in the pain journey map 3 based on the reported pain level and timeline, which could help us recognize the most common abnormal activity when pain occurred to indicate the presence of pain by the bio-sensor.

According to all the caregivers, the patients get up in the morning and have lunch at noon. As they have chronic pain in their leg and the effect of AD, they are not interested in activities that need much effort so they sit somewhere in the house (e.g., sofa or bed) for hours till night. However, when they are unwell some day, they may start to walk around the house after lunch. Furthermore, they cannot stay still and fall asleep at night. Instead, they will get up and go to the toilet repeatedly. F1, F3, F5, and F6 caregivers mentioned:” We did not know why my grandma/grandpa suddenly became an active walker at first, and she only walked back and forth in the room, till we asked her again and again and finally knew she is in pain.”

In general, our interview showed that the abnormal physical activity was extra walking frequency, which could be a potential parameter to detect to represent the pain occurrence. We can use accelerometers to collect these bio-data. When there is abnormal physical activity, the physiological signal sensors could give us real-time feedback from the patient if there is any pain condition.

### 3.3 Analyzing the presentation of pain data and the interaction between Stakeholders

The accelerometer needs to be carried around for real-time data collection, which means it needs to be embedded in a wearable device. Hence, **the third goal of our interview was to probe what form of the wearable device would be acceptable for the patient to carry, and how to make the pain information perceived by caregivers since the wearable is on the patient**. In addition, we realized that the communication process should be two-way, including transmitting information and receiving feedback. As F1, F3, F5 and F6 said, “When the patient was in pain, he/she would do some strange things, like screaming and blaming, in my opinion, they were trying to attract my attention to them … Sometimes I came along and did something or even just talked to him/her, he/she seemed better … ”. Thus, only informing the caregiver of the presence of pain is not a complete pain communication process for the patient; they also need to feel the feedback from the caregiver.

Therefore, we wanted to record and analyze the patient’s daily living environment, their reliable daily objects and access to them, and the caregiver’s approach to pain relief when they find the patient is in pain. They all consented to tell their stories and allow the recording. However, due to the COVID-19 safety restrictions, we could not record at their home in person. Therefore, we asked the caregiver to record the above information in a diary, including images and text recordings of the environment and actions.

From descriptions from caregivers and sharing of leisure records, we identified the following insights and design criteria that can define our design.

**FIGURE 3:**
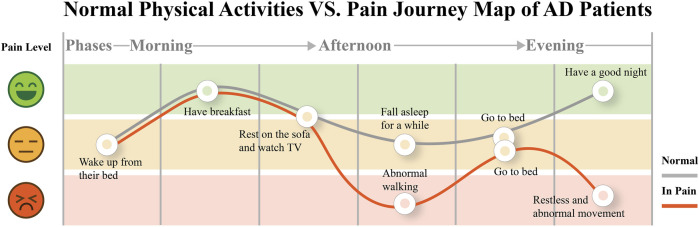
Pain journey map of AD patients. They usually conduct quiet physical activities in daily life. However, when they are in pain, abnormal walking frequency (indicated as much more walking steps than usual) will appear in their leisure.

1) **DC1: The accelerometer sensor can be the potential bio-sensor for tracking the gait data, which will indicate the pain of patients.** Figure 3 indicates that patients tend to stay silent or sit still on the sofa almost all day. While they are in pain, the frequency of walking will become high, and they will be more restless during the day and night. F1 told us, “My grandma always wanders around the house, but we just know that she was in pain long after she was in severe pain and walked anxiously … “, F6 said, “My grandfather will get up and walk around many times at night when he feels bad … “.
2) **DC2: The tangible interaction interface should be portable and of a soft texture that can be easily held in hands.** Almost all older adults show resistance to wearable products, but we found that the elderly prefer to hold some small objects (such as tissues, clothes, handkerchiefs, and toys). F3 told us, “We made my grandma a box containing some tissues and cloths, in case she can grab them and fold them beside her sometimes”, the others said, “A handkerchief is always in his pocket … he would take it out sometimes and just held it … ” From time to time, the patients will play with the objects in their hands. F1,2,3,4,6 indicated to us that the patients are resistant some wearable objects that closely attached to their bodies. “My aunt bought my grandma a lot of jewelry, but every time we let my grandma to try on something, she just took it off quickly” said by F1. “We once bought the patients a smart watch in case he/she got lost, because sometime he/she went out by himself/herself. But he/she did not like to put on the device, so the watch was left alone now.” mentioned by F5 and F6. These evidence help to exclude the bio-sensors that need to adhered on user’s skins, e.g., the ECG/GSR sensors.
3) **DC3: The tangible interaction needs to capture the attention of the older adults, presumably by using visually or auditorily salient features.** It is difficult for caregivers to know from the patient’s abnormal physical activities whether the patient is in pain, unless the patient starts to make a loud noise to attract attention. F3 suggested, “My grandmother’s legs hurt for no reason … we always call her name loudly and talk to her when she is about to cry, she seems to forget that she is in pain.“, and F6 shared, “We will bring out my grandpa’s er’hu, a traditional Chinese instrument … As he sees the er’hu, he will stop being mad for his pain.” The comforting method caregivers use is to make some attractions like newfangled gadgets or talk about other things loudly to divert the patient’s attention so that the patient can be relieved.
4) **DC4: The tangible interaction can follow some existing therapy, which allows the caregivers to follow existing approaches in pain management.** F2 mentioned, “It is difficult to summarize what method can make my grandmother becomes soothing. Sometimes gently patting her will be more useful, but sometimes it is more effective to talk to her.” F5 told us, “Because the patient does not particularly like anything, we can only try to meet her needs. For example, sometimes we will help her to find some food.” The facts suggest that it is often difficult for caregivers to have a general idea of communicating with and helping the patient, especially if the patient is impatient. Therefore, the rules of certified therapy, e.g., MSS therapy and music therapy, can be an effective mechanism for interaction for caregivers to engage in pain communication with the patients. For example, in MSS therapy, they shall provide a fidget tool to the patients to guide them to interact with sensory patterns in a certain order.

### 3.4 The user persona

**FIGURE 4:**
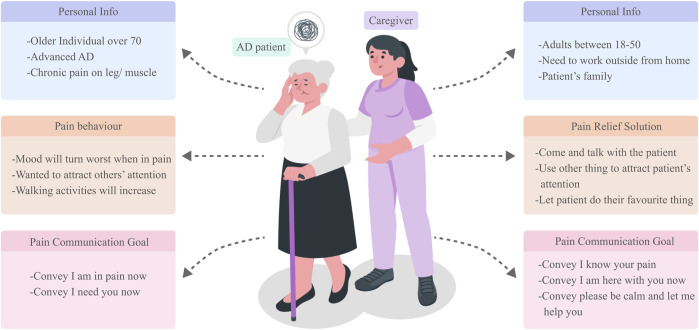
User persona of AD patient with chronic pain and their caregivers.

Based on our interview insights, we constructed user personas for our design, representing both AD patients and their caregivers, as illustrated in Figure 4. These personas serve as a continuous reminder of our users’ primary needs and critical challenges throughout the design process. Our focus centers on AD patients aged over 70, in advanced AD stages, and experiencing chronic leg and muscle pain. Their pain-related behaviors underscore an essential need for support and attention, particularly from family and caregivers, to alleviate their discomfort. Correspondingly, our caregiver persona is an individual aged between 18 and 50, deemed more capable of attending to the needs of such patients. Often working remotely or away from the patient, these caregivers find value in consistent patient accompaniment, regular feedback, and employing diversions to engage the patient’s attention. Their overarching goal in pain communication is to promptly discern the patient’s discomfort and ensure holistic wellbeing, encompassing both physical and psychological dimensions.

## 4 E-textile system construction

### 4.1 Pain communication handkerchief framework

#### 4.1.1 Presentation rationale

As discussed in Section 3.1, our primary goal was to communicate the existence or absence of pain to caregivers. When patients experienced pain, an indication would appear on the caregiver’s end. Integrating a bio-sensor with a soft-textured item led us to select e-textiles as the medium. We adopted the handkerchief form due to its familiarity and acceptability to patients for interaction. Guided by DC4, we utilized MSS therapy to direct the interactive feedback for caregivers. The caregiver’s interactive handkerchief was designed to act as an actuator, triggering a multi-sensory response on the patient’s handkerchief. For instance, when a caregiver activated a floral button on their handkerchief to initiate light stimulation, a corresponding colored light illuminated on the patient’s side to capture their attention. Furthermore, the caregiver’s handkerchief acted as a reference during interactive matching tasks, grounded in cognitive stimulation therapy’s matching task principles, which require the caregiver’s end to have a similar representation to the patient’s end.

#### 4.1.2 System framework design

**FIGURE 5:**
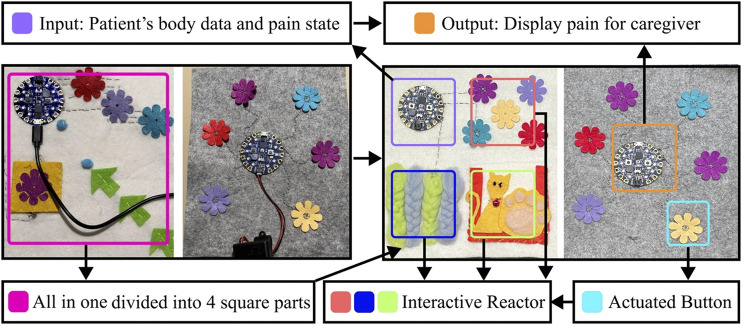
(Left) is the first design of the e-textile handkerchiefs. After tried out with the patient and the caregivers, the handkerchiefs are iterated like the right.

**FIGURE 6:**
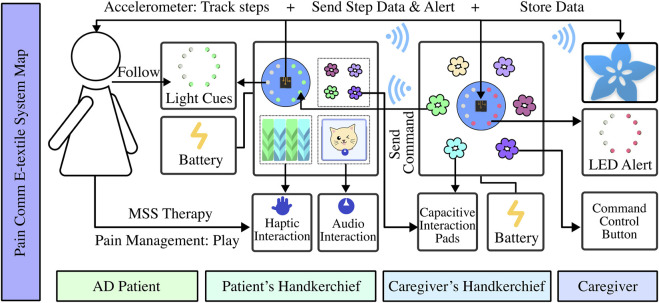
System construction of paired pain communication handkerchief. The patient’s part consists of a processor board with an accelerometer sensor, a pressure sensor flower that can detect pressure, wool ropes connected to capacitive touchpads on the board, and a speaker that can play music. The caregiver’s part includes a processor board that shows light alerts and some controller-touched flowers that can send different game commands.

As shown in Figure 5, the e-textile was composed of four main parts: the input, which monitored the pain state and transmitted the data to the e-textile part of the caregiver; the output, to display pain data to the caregiver for purposes of informing pain occurring in the patient by changes in the displayed state of this electric device on the textile; actuator button, it allowed caregivers to provide feedback with the system, and also the guide to the patient to interact with the e-textile; interactive reactors, which could let the patients follow the guide of the caregiver to touch and explore with the materials. Bio-sensors implemented on the input side were the accelerometer, which could capture chronic pain AD patients’ real-time physiological data, reflecting the user’s comparative physical activities condition directly and pain condition indirectly. Red LEDs on the caregiver’s CPB were displayed as the output of the pain condition for communication and alert purposes. As shown in Figure 6, after the pain was detected, the CPB of the patient’s side sent a signal to the caregiver’s side through a Bluetooth module. The bio-data was sent to and stored in CPB’s mobile APP simultaneously. This APP could be a backup interface for caregivers to learn about the pain data and control the handkerchief. The actuator buttons (conductive fabrics) were for the caregivers and patients to interact with their hands, controlling the device and the LEDs as the pain and feedback indicators. When caregivers recognized the alert signal, they could press the button on the CPB to activate the feedback mode. In feedback mode, they could choose different cloth buttons. Each button could control one sensory interaction on the patient’s side through the Bluetooth module. For example, if the blue button is pressed on the caregiver’s side, the blue LEDs will light up to guide the patient to touch the blue flower; if the yellow one is pressed, the sound could be triggered on the patient’s side.

#### 4.1.3 Technical Implementation

During the implementation, we employed the Adafruit Circuit Playground Bluefruit (CPB) to gather patient biofeedback data. The CPB is the main electronic board that processes and then presents the display data on both sides. We use the three-axis accelerometer lis3dh on the Adafruit CPB, which can collect the three components of the Alzheimer’s patient’s motion (forward: roll, vertical: yaw, and sideways: pitch), corresponding to the acceleration in the *x*, *y*, and *z*-axes. The data collected will be used for making a steps calculator and physical activities tracker. The calculation method is shown below:

$$SVM_{\text{acc}}\left(k\right)=\sqrt{a_{x}{\left(k\right)}^{2}+a_{y}{\left(k\right)}^{2}+a_{z}{\left(k\right)}^{2}}$$ (1)

*K* is the time step, *ax*(*K*), *ay*(*K*), *az*(*K*) are the filtered accelerations of the *x*, *y*, and *z*-axes of the three-axis accelerometer.

### 4.2 Attachments design details

As illustrated in Figure 6, we integrated interactive patterns, akin to the fidget blanket, into the handkerchief for therapeutic engagement. Informed by DC3, we incorporated luminous floral patterns for visual engagement. Observing patients’ tendencies to fold and manipulate soft objects during pain episodes, we introduced a folding mechanism with arrows to guide patients to fold the handkerchief from one side to the other side. Additionally, inspired by MSS therapy’s fidget blanket, which encourages task completion, we added a pocket with a retrievable flower for patient to seek the flower and replace it. To facilitate guided interactions, a handkerchief equipped with pressable floral buttons was designed for caregivers. The color-coded system between caregiver and patient handkerchiefs enabled synchronized interactions upon button activation.

### 4.3 Tangible interaction with MSS therapy

**FIGURE 7:**
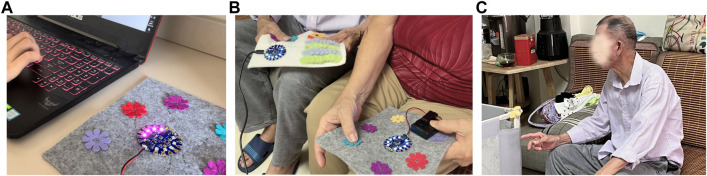
**(A)** When the patient is in pain detected by the sensor but the caregiver is concentrating on his/her own work, the processing board on the patient’s side will send a signal to the caregiver’s side, triggering the red light on to alert the caregiver **(B)** The caregiver comes to the patient when the patient is in pain. The caregiver presses a blue flower button to emit the blue light on the patient’s handkerchief, and the patient can follow to experience the MSS therapy. **(C)** The patient is humming while he presses the sound interaction pattern.

When the patients were in pain, and the number of steps exceeded a threshold, the caregiver’s handkerchief received a signal to light up a red light indicating abnormal pain. At this time, the caregiver could come to the patient and control the handkerchief with interactive patterns to send guidance commands, implementing the MSS therapy. Thus, the caregiver could press the flower with the same color as the ones on the patient’s handkerchief, emitting the same color lights on the patient’s handkerchief or triggering the music and sounds. To complete the task, the patient did multi-sensory stimulation therapy by following the caregiver’s guidance to perform simple matching tasks using the handkerchiefs with interactive sensory components (such as pressing the red flower when the red light is on). We sew touch points connected to capacitive touchpads on the CPB on the tactile wool rope, which also light up the handkerchief when the patient strokes the rope. The procedure is shown in Figure 7.

## 5 Evaluation: Case study

### 5.1 Design exploration and iteration

Due to the health concerns for the participants during COVID-19, we only invite one family (F6) to try out our prototype in person. F6 is also the most typical and important user case that matches our user persona and could provide valuable feedback for our design. After the presentation, we interviewed the caregivers. When the patient was in pain and the pain alert was triggered, the caregiver came by and triggered the multi-sensory stimulation therapy, leading the patient to calm down. The patient was a bit confused at first, but got calmer and learned how to use the system as feel accompanied by his family. The caregiver then taught the patient the way to interact. They tried a few times using the prototype, and when we asked the patient how he felt, he told us he was happy. For the caregiver’s handkerchief, she reported that the alert lights let them know the pain status quickly, and that the selection of flowers to control sensory interaction was easy to use. However, when patients used the handkerchiefs, they could correspond to the colors of the flowers to interact with and the blinking lights, but it took much work to know how the arrows and pockets interacted. Caregiver reported similarly that the arrows might no longer be recognizable to patients at this stage; they also suggested replacing them with small animals or flowers, as older adults are more attracted to these patterns. The task of finding and placing the flowers could be further simplified, similar to touching the flowers corresponding to the light. The caregivers also suggested that the existing interaction could be enriched. For example, sound could be added to the design since older people love to listen to old-school music or opera songs.

**FIGURE 8:**
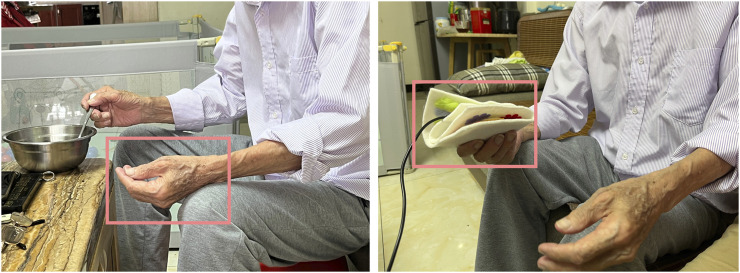
The patient is used to holding his hand. When he got the handkerchief, he naturally hold it without resistance.

According to the feedback from our participants, we first changed the pattern layout into a square arrangement as the patient loved to fold their handkerchiefs neatly, as shown in Figure 8. We changed the panel of seeking small flowers to woven wool stripes to give patients a better tactile experience. We also replaced the part of the arrow with a kitten and added a sound module. When the patient strokes the kitten, the kitten will make a sound to enrich the multisensory experience. There is a hidden pocket behind the kitten to hold the sound module, which can also be a replacement for the simple seeking task.

### 5.2 Design evaluation and results

We engaged F6 and another caregiver (labeled as C1) from an Alzheimer’s Disease (AD) care center in Hong Kong, who also provided home care services, to assess our final design. With their consent, we observed and interviewed them in a domestic setting. A day prior to the interview, we met the caregivers and demonstrated the handkerchief’s usage, subsequently handing it over to record the patients’ physical activity. To validate the device’s effectiveness in pain detection, we juxtaposed the device’s pain alerts with the caregivers’ self-recorded observations of the patients’ painful behaviors. Our hypothesis was that a temporal correlation between the patient’s exhibited pain symptoms and the device’s alerts would attest to the system’s utility. We told the caregiver first to ask the patient if he/she would like to hold the handkerchief or put it in his/her pocket. The handkerchief would be placed in the patient’s pocket for the rest of the day with consent. We visited the patient in the late afternoon, the frequent pain period of the patients’ day, and introduced the caregivers to the method of providing instant feedback with the handkerchief. The patients followed their daily activities and interacted several times using the handkerchiefs when it suggested the presence of pain to the caregiver. After the patients were reassured and his status was stable, we conduct a semi-structured interview with the caregivers to learn about their experience interacting with our system. The interview framework drew inspiration from our research questions and the User Engagement Scale (UES) (O’Brien et al., 2018), addressing the following domains: 1) Perceived Usability: How did the system assist in discerning pain presence? Were there instances of confusion or difficulties in pain perception using the system? 2) Felt Involvement: How did the system facilitate both caregiver and patient engagement in pain communication and MSS feedback? 3) Endurability and Novelty: What elements of the system maintained your engagement? Which features made it compelling for continued use, and which aspects warrant improvement?

#### 5.2.1 Pain Perceived Effectiveness for the Caregiver

A red light represents a warning in line with our common sense, and a sudden light can be noticeable to draw the caregiver’s attention. During the test, the caregiver F6 was cooking on the other side of the house and put the handkerchief somewhere noticeable. “When the lights are on, I know something is happening on the patient’s side. Previously, I had to keep yelling out to him to confirm his condition, sometimes without getting any answer.” The prompting using only lights still needs to be enhanced. The caregiver F6 told us, “I think it would be more effective if it vibrated like an alarm, because sometimes I might miss the reminder, just like I miss a message.” Alerting caregivers in multi-channel way and multi times may improve the ability of the pain communication of this system.

#### 5.2.2 Involvement in the Communication for Both Patients and Caregivers

When patients felt pain, they always squeezed the handkerchief, and their facial expressions became serious. However, when the caregiver interacted with them, they became visibly happy and even initiatively expressed to us that they were happy. We personalized a piece of the F6 patient’s favorite music in the sound effects, and the patient gradually relaxed and hummed along with the music after the sound was triggered, as seen in Figure 4. The patients seemed more interested and sensitive to the sound component, and could feel engaged in the soft and relaxed atmosphere. The caregiver F6 explained, “This handkerchief allowed me to give faster feedback to my grandpa. I think immediate companionship is important for them. They are just like children now. When they feel uncomfortable and are cared for, they may feel happier, and the pain level feels reduced to some extent.” Besides being involved in the therapy to heal their painful feelings, the most significant effect of our system is that both the patients and caregivers were more involved in a closer family companionship with effective pain communication. C1 indicated that although they could not have more interactions besides guiding the patients to play with the MSS handkerchief, they still felt involved because the patient gave positive feedback like a happy face or calming down, which shows that the intervention is helpful. However, C1 suggested, “I had an experience making music with an instrument with the patient to do the MSS therapy before, and we both enjoyed the process of playing and can share a sense of accomplishment because we got some interesting outcomes together”. If caregivers can also join the play process and share enjoyable experiences together with the patients, it would be more encouraging to use the system.

#### 5.2.3 Activity and Pain Tracking

**FIGURE 9:**
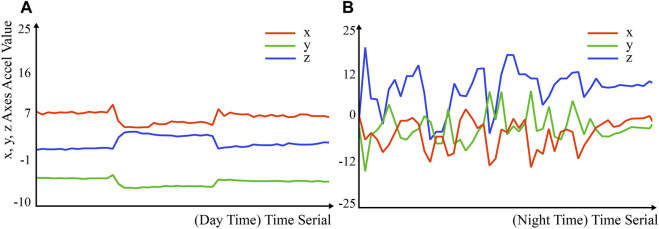
**(A)** Accelerometer data in the day when AD patient are not in pain. **(B)** High intensity of physical activities at night. “Accel” represents acceleration.

The data from the accelerometer, presented in Figure 9, along with caregiver feedback, suggested that heightened physical activity correlated with the patient’s anxiety and severe coughing spells late into the night. An alignment between the system’s alert times and caregiver-reported pain episodes, predominantly around 7 p.m., insinuated the system’s capability to detect and communicate pain. The patient quickly became calm after the repetitive interaction with the handkerchief. We suspected the patient’s pain level was not high in this case. The caregiver F6 also confirmed it in the interview: “When he is very uncomfortable, he usually curses, his temper will be terrible, and he simply cannot calm down.” To some extent, the patients’ patience could reflect their pain and emotional state.

#### 5.2.4 Endurability and Engagement for the Caregiver

The novelty of our design seemed very high to the caregiver. However, the entertaining feature of the MSS therapy was a bit low for adults with normal cognition, leading to low endurance and felt involvement for the caregiver. This directly affects the caregiver’s patience and trust in the design. As the caregiver F6 said, “The gameplay was too simple for me. It is great that I can use it to accompany the patient, but I gradually got bored. I would have been surprised if the gadgets were more varied.” This inspires us to make each fidget pattern changeable, turning-based handkerchief sewed with a processor into a blank board, on which we can constantly update and iterate the interactive components. For the patient, engagement comes from being constantly challenged and taught to do the task, and receiving some encouraging feedback after completing the task, which was previously difficult to get because the patient was usually not asked to do anything resulting in less interaction between the patient and caregiver. (In accordance with the patient’s expression in the test and the caregiver’s feedback - the patient rarely had the patience to do the task alone before, even if he was invited to play his favorite er’hu song, but today the patient seems much happier and patient.) The caregiver C1 shared another perspectives: “As I always conduct the simple tasks with simple tools in the MSS therapy, this system seemed is similar to those tools I used before. However, my engagement came from stories sharing of the patients. For example, I will ask the patients about their experience with the objects that we are interacting with, and the most of patients are very willing to share stories with me.” Inspired by this, designing the interactive attachments that are relate more on the daily life may help to enhance the engagement for both the patients and caregivers.

## 6 Discussion

To address the research question concerning differences in pain communication needs between patients and caregivers, we observed patients’ daily lives and their pain expression capabilities and conducted in-depth interviews with caregivers. Our findings indicated that the most pressing communication needs for both parties lies in different stages of communication. This challenges conventional understanding of the pain process, typically similar to a physician’s diagnostic approach. Past perceptions of pain communication have led to designs primarily focused on patients conveying pain to caregivers, overlooking the caregiver’s need for effective feedback communication. In our study, we indicated the two different needs of communication and propose a two-way pain communication framework.

To discern and measure patients’ pain through non-verbal behavior, we tracked patients’ daily activities and abnormal behavior during pain episodes to match possible bio-sensor detection capabilities. Abnormal walking patterns among patients were particularly noticeable. Based on previous pain detection device research and clinical evidence linking AD patients’ pain with abnormal walking, we incorporated accelerometers to aid in non-verbal pain behavior detection. In testing, we found this method could provide insights into a patient’s overall daily pain condition. Align with Caregivers’ reports that patients’ adverse moods and behaviors typically presented after accumulated pain reached a certain threshold, which our device might potentially map this way of pain expression. However, such a detection method has significant limitations, like missing momentary pain spikes.

To address the final research question on aiding caregivers in feedback and interaction, we introduced MSS therapy and e-textile devices resembling fidget blankets. On one hand, therapy procedures and toy interactions guide caregivers in leading patients into cognitive stimulation through play (Nevay et al. (2017; 2019)). On the other hand, tangible e-textile tools offer a means to transform verbal exchanges into playful communication, not only diverting patients’ attention to calm them but also granting caregivers an avenue to access communication.

## 7 Limitation and future work

In the design process, the diversity and number of user population limit our designs and findings. F6 is the only family that got involved in the participatory design process in person. And we only got two groups ofparticipants in our analysis process. Though these use case is quite typical to our user portrait and has provided many valuable insights to our design, to validate the pain detection ability of the system, more AD patients with chronic pain and their caregivers from different backgrounds should join the design process to evaluate the prototype better and general to use. We may also recruit some families from the Western culture in case there may be different attitudes in pain communication. Moreover, we only discuss the design of the pain information display based on one usage scenario (working scenario) of the caregiver. More scenarios still need to be explored because different usage environments will affect the effectiveness of the way information is displayed. Different presentation modes can be selected through buttons by the caregivers. Thus, they can easily switch the notification form considering their condition, just like turning off the ringing mode on the smartphone in a meeting but turning it on when in need of an alarm to notice the message immediately.

For the design of the handkerchief, wireless and smaller battery may help to improve user acceptance. The enrichment of the interavtive components will limit the interest and engaged level when people use it. More display modes can be prototyped to communicate more information about pain. For example, different lighting conditions (number of lights, color, intensity of light, lighting pattern) may represent different pain features. However, a single visual effect is less suggestive. Multimodal cues allow more effective pain communication in the future. Like the fidget blanket, the pain communication handkerchief can be personalized and iterated to make the MSS interactive parts relevant to each user’s preferences. Music is among the MSS interaction components that create more significant concerns for both caregivers and patients. In previous non-pharmacological pain management interventions, music has repeatedly been proven effective for pain-relief (Park, 2010; Bao and Landers, 2022). Different music links to the specific memory in AD patients, creating a sense of familiarity and mental relaxation (Gerdner and Schoenfelder, 2010). Music can also be customized to make the design more relevant. In the future, we can make a digital interface for the caregivers to choose and upload the favorite music of the patients into the central processing board. Play and stop functions will also be designed correspondingly in MSS patterns so patients can switch the songs anytime. Secondly, the Bluetooth connection of the patient’s and caregiver’s handkerchief causes distance limitations. In the future, a wifi-based system need to be integrated like other processing board supported wifi connection and mobile APP to ensure the distant communication between devices, and simultaneously enhance the complexitivity of caregiver’s devices.

For pain recognition, only using one kind of bio-sensor is not precise enough. Multiple sensors in one system can improve accuracy. We need to consider wearing comfort and detection efficiency to adopt multiple sensors. With sufficient participants and data collecting duration, the accuracy of the sensors could have been better measured and compared longitudinally. Besides, adding the electronic component brought inconvenience and unnaturalness in use, such as increased weight and hardness, even if the interaction modality is enriched. Finally, the current premise is that if the number of steps by a patient exceeds a threshold (over 8k steps), that could be due to pain-related behavior. However, the step threshold represents the overall pain of the day, which may result in some lag in pain detection. For instance, if the patient starts walking due to pain in the morning or at night when the threshold is not hit, the system may delay telling the caregiver about the condition. In this circumstance, other compensating sensors like GSR should activate to detect the stress and pain condition for pain report. Machine learning techniques (Bai et al., 2021; Bai et al., 2022) may also be employed to help the system learn the pain feature of particular patients, enabling more real-time feedback when the pain features appear in patients’ daily activities.

For the user study and analysis, the period of testing the prototype was not sufficient, so in the future, we could adopt the experience diary to help caregivers record the user experience for a more extended period to provide us with more valuable reflections on user behavior. We can also challenge the patients by repeating to play with the prototypes for times to test the relation between persistence ability and pain level to provide more insights into the design.

## 8 Conclusion

Older adults suffering from AD and chronic pain have trouble expressing their pain, leading to issues like being untreated or mistreated by their caregivers and medical professionals. The bio-feedback and e-textile technology can help them to track and monitor their body condition and report to themselves and others in a user-friendly and comfortable interface. This e-textile handkerchief can be a potential alternative approach as two-way communication between non-patients and AD patients. For one thing, it reflects AD patients’ temporal pain condition, Sending out the pain message objectively without less bias and uncertainty, compared with the previous approached (Zwakhalen et al., 2007). For another, caregivers can find a path to provide feedback without worrying about misunderstanding, or lack of experience before. The system provides a possibility for caregivers to give feedback on the patient’s help message. As the caregivers’ consensus, AD patients need people’s company to feel safe and live happily. However, when communication is hindered, many people do not know the appropriate way to accompany their AD family, especially when the patient is in pain and distress. A handkerchief can be a natural and tangible user interface for an AD patient, because this form of e-textile does not violate the patient’s unconscious behaviors and habits.

Our design enlightened caregivers about the potential of e-textile devices in aiding the understanding of a patient’s pain status, possibly fueling interest in a broader range of MSS tools and methodologies, as suggested by F6. However, during the design phase, it is evident that merely focusing on the patient-centric features and interactions is insufficient. Just as we account for the distinct pain communication needs of patients and caregivers, the requirements for interaction should be tailored based on cognitive levels to enhance the user experience.

## Data Availability

The raw data supporting the conclusion of this article will be made available by the authors, without undue reservation.
